# Iron Porphyrin as a Cytochrome P450 Model for the Degradation of Dye

**DOI:** 10.3390/molecules27227948

**Published:** 2022-11-17

**Authors:** Dan-Dan Ren, Xiaoyan Lu, Li-Ping Zhou, Huanghongjun Tian, Shuang Wang, Lu-Fang Ma, Dong-Sheng Li

**Affiliations:** 1 College of Materials and Chemical Engineering, China Three Gorges University, Yichang 443002, China; 2 Henan Key Laboratory of Function-Oriented Porous Materials, College of Chemistry and Chemical Engineering, Luoyang Normal University, Luoyang 471934, China

**Keywords:** iron porphyrins, dye degradation, gentian violet, iodosylbenzene, UV–vis

## Abstract

Organic dyes are widely used in the textile, biological, medical and other fields. However, a serious environmental problem has appeared because of the presence of organic dyes in industrial aqueous effluents. Thus, the efficient treatment of organic dyes in industrial wastewaters is currently in real demand. The current study investigated the oxidative degradation of the organic dye gentian violet by *meso*-tetra(carboxyphenyl) porphyriniron(III), [Fe^III^(TCPP)] as a cytochrome P450 model and iodosylbenzene (PhIO) as an oxidant at room temperature. The degradation reaction was monitored by UV–vis absorption spectroscopy via the observation of UV–vis spectral changes of the gentian violet. The results showed that the efficiency of catalyzed degradation reached more than 90% in 1 h, indicating the remarkable oxidative degradation capacity of the [Fe^III^(TCPP)]/PhIO system, which provided an efficient approach for the treatment of dyeing wastewater.

## 1. Introduction

As is well known, dyes are widely used in the papermaking, textile, food, cosmetics and other industries [1,2]. With the increase in material and cultural needs, the dye industry continues to develop and grow. About 300–400 million tons of industrial waste are discharged into rivers every year [3,4]. These persistent organic dyes found in industrial aqueous effluents can accumulate in the body after entering the human system and result in serious health problems in the cardiovascular, endocrine, nervous and immune systems, which may even be passed to the next generation. Among the various dyes, triphenylmethane dyes, for example gentian violet, are the largest and most versatile, and they play a predominant role in human and veterinary medicine and the textile processing industry [5]. However, gentian violet is difficult to degrade and can remain in the environment for a long period. It is toxic to aquatic and terrestrial life, and it is considered a mitotic poisoning agent and a potent clastogene [6]. Thus, the efficient treatment of gentian violet in industrial wastewaters is currently in real demand [7].

In the past few decades, people have invested a lot of energy into the treatment of dye-containing effluents [8,9,10,11,12,13,14,15,16]. Various methods to degrade dyes, including chemical [17,18], physical [19,20,21,22] and biological [23,24] methods, have been developed and widely applied for the degradation of organic dyes. However, these traditional methods have certain limitations. For example, air-bubbling visible-light photocatalytic degradation can eliminate pollutants in wastewater under mild conditions [25,26,27]. However, due to the small active area of photocatalysts, insufficient light absorption and instantaneous electron hole separation, the photocatalytic degradation activity of this method is reduced. In addition, adsorption is the most common technology used to remove pollution from textile, printing and dyeing industrial wastewater [28,29], but the adsorption capacity of this method is generally low, and new adsorbents are still being developed. Another example is the microbial treatment of printing and dyeing wastewater, which is considered to be an economical and feasible method without secondary pollution, yet biodegradation has the disadvantages of it being unstable and time-consuming. Thus, it is urgent and necessary to develop new methods to degrade dye-containing wastewater.

Enzymes are among the most sophisticated catalysts due to their unparalleled catalytic specificity and efficiency. For example, cytochrome P450 is able to catalyze a variety of oxidation reactions, including the oxygenation of aromatics, hydroxylation of C–H bonds and epoxidation of double bonds at room temperature and atmospheric pressure [30]. However, due to their high cost, highly restricted catalytic conditions and fragile nature, the practical applications of enzymes have been hindered. To overcome these problems of enzymes for practical applications, biomimetic systems have subsequently emerged as an effective approach to synthesize highly efficient biomimetic catalysts via mimicking certain key features of enzymes [31,32].

Metalloporphyrins, as a representative model of cytochrome P450, have been extensively studied, in which a number of metalloporphyrins have been used to catalyze the degradation of dyes [33,34,35,36,37,38,39,40,41]. For example, manganese porphyrins, manganese tetra-(*p*-carboxylphenyl) porphyrin (MnP(COOH)_4_) and manganese tetra-(*p*-methylpyridium) porphyrin [Mn(TMPyP)], among others, have been used to catalyze the decolorization of azo dyes with H_2_O_2_ as an oxidant in the presence of imidazole in aqueous solution and in nonaqueous solvents [40,41,42]. Meyer and coworkers investigated the oxidative degradation of dyes by *meso*-tetrakis(1-methylpyridinium-4-yl)porphyrinatomanganese(III), [Mn^III^(tmpyp)], in aqueous solution, which showed that it is an efficient catalysts for the oxidation of various substrates, and high-oxidation-state oxomanganese(IV) and oxomanganese(V) intermediates have been proposed to play a significant role in these reactions [39,43,44,45,46,47,48,49,50,51]. Very recently, Meyer and coworkers also reported an iron porphyrin, *meso*-tetrakis(1-methylpyridinium-4-yl)prophyrinatoiron(III) ([Fe^III^(tmpyp)]) as an efficient catalyst to degrade azo dyes using *meta*-chloroperoxy benzoic acid (*m*-CPBA) as an oxidant in aqueous solution at room temperature [52]. They found that the dye degradation rate was determined by the concentrations of [Fe^III^(tmpyp)], *m*-CPBA, dye and surfactants and the pH value of the solution, and they suggested that [Fe^III^(tmpyp)] is transformed into a high-valent iron(IV)-oxo porphyrin π-cation radical species [Fe^IV^(O)(tmpyp^+•^)], referred to as compound I (Cpd-I), within 20–30 ms followed by the formation of relatively stable [Fe^IV^(O)(tmpyp)], referred to as compound II (Cpd-II) by spectral analyses and kinetic data. However, very limited ironporphyrins used as cytochrome P450 models have been reported to catalyze the oxidative degradation of dyes [35,36,38,42].

**Figure 1:**
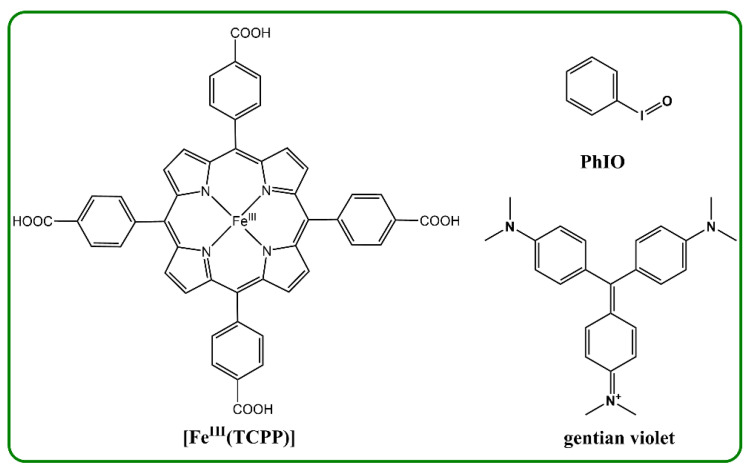
Structures of the catalyst [Fe^III^(TCPP)], the dye gentian violet and the oxidant iodosylbenzene (PhIO) used in this study.

The ironporphyrin, 5,10,15,20-tetra(4-carboxyphenyl)porphyrinato iron(III) chloride, [Fe^III^(TCPP)], as a cytochrome P450 model, has been reported to be grafted in metal–organic frameworks, which have been widely applied in the fields of photodynamic therapy, signal probing, photocatalytic degradation, the photocatalytic reduction of carbon dioxide and the selective reduction of nitrogen to ammonia [53,54,55,56,57,58]. However, the degradation of dyes by [Fe^III^(TCPP)] has rarely been studied [52,59]. Herein, we investigated [Fe^III^(TCPP)] as a cytochrome P450 model for the oxidative degradation of a selected dye, gentian violet, employing iodosylbenzene (PhIO) as the oxidant (Figure 1) in methanol at 30 ℃. The degradation reaction of gentian violet was monitored by UV–vis absorption spectroscopy via the observation of UV–vis spectral changes in the gentian violet. The [Fe^III^(TCPP)]/PhIO system gave a remarkable oxidative degradation of gentian violet.

## 2. Materials and Methods

### 2.1. Materials and Instruments

All reagents and solvents were commercially purchased and used without further purification. Dichloromethane (DCM), methanol and sodium hydroxide (NaOH) were obtained from Damao Chemical Reagent Factory), Tianjin, China. Ethyl ether (Luoyang Chemical Reagent Factory, Luoyang, China), gen-tian violet (Tianjin Guangfu Fine Chemical Research Institute, Tianjin, China) and 5,10,15,20-tetra(4-carboxyphenyl)porphyrinato iron(III) chloride ([Fe^III^(TCPP)] (Bide Pharmatech Ltd., Shanghai, China) were used as provided. (Diacetoxyiodo)benzene (PhI(OAc)_2_) and 3-Chloroperoxybenzoic acid (*m*-CPBA) were provided by Shanghai Macklin Biochemical Co., Ltd., Shanghai, China. The water used in all experiments was distilled water. UV–vis absorption spectra were recorded on a Hitachi U-3010 spectrometer.

### 2.2. Preparation of Iodosylbenzene (PhIO)

Iodosylbenzene (PhIO) was prepared by a previously reported method [60]. A total of 8 g of (diacetoxyiodo)benzene (PhI(OAc)_2_) was weighed and placed in a 250 mL beaker and wrapped in tin foil for light protection. A total of 4.5 g of sodium hydroxide (NaOH) was weighed in a 50 mL beaker and dissolved with 30 mL H_2_O. The NaOH solution was slowly added to the beaker containing the solution of PhI(OAc)_2_ within 0.5 h, which was then stirred vigorously at room temperature for 1 h. Then, 20 mL H_2_O was added and continuously stirred for 1.5 h. The resulting solution was then filtered and washed with water until the pH value of filtrate reached 7, and then the precipitate was washed with dichloromethane and ethyl ether, respectively, dried and kept in a refrigerator for further use. Its purity was periodically controlled by iodometric titration [61].

### 2.3. Determination of Concentration of Gentian Violet

**Figure 2:**
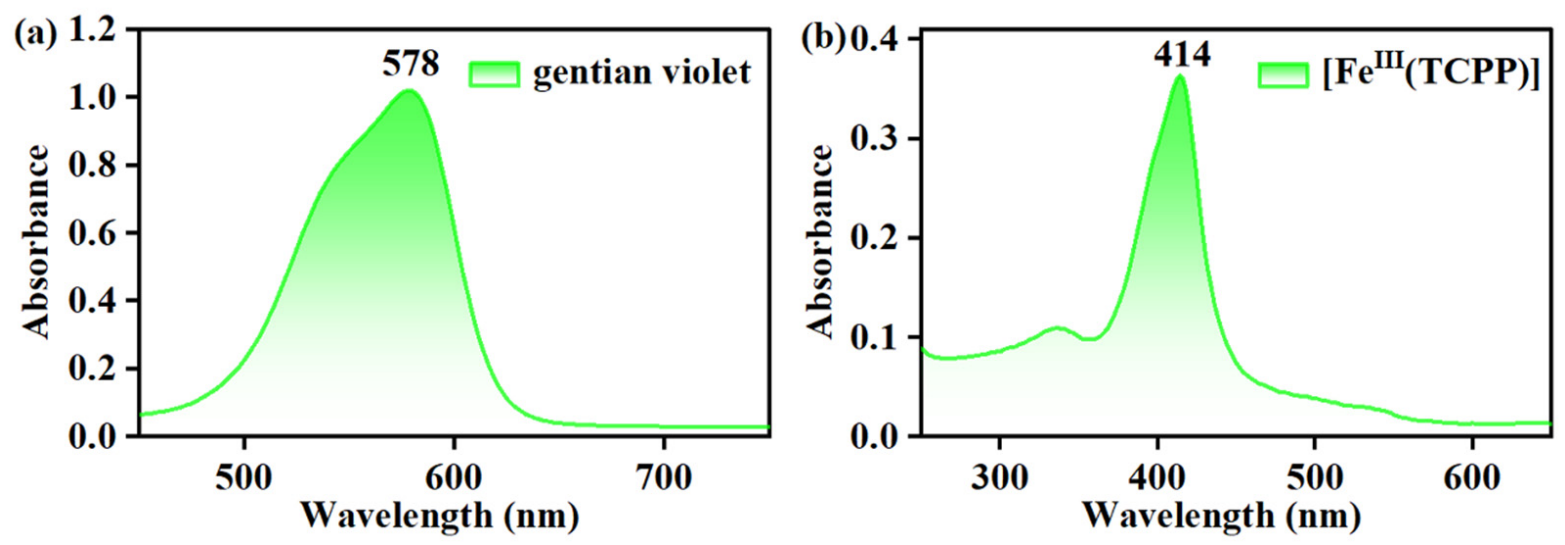
UV–vis spectra of gentian violet (**a**) and [Fe^III^(TCPP)] in MeOH (**b**).

The concentration of gentian violet in the resulting solution was determined by a UV–vis spectrophotometer with a quartz cuvette (path length = 10 mm) in the wavelength range of 200–800 nm. The UV–vis spectrum of 0.05 mM gentian violet in MeOH exhibited a distinct absorption band at 578 nm (*λ*_max_) with an absorbance of 1.022 (Figure 2a), and its extinction coefficient (*ε*) was then calculated to be 20,440 M^−1^ cm^−1^ using Beer’s law, *A* = *ε*c, where *A* is the absorbance at 578 nm and c is the concentration of gentian violet. Thus, the concentration of gentian violet in the reaction solution could be determined from the absorption band at *λ*_max_ = 578 nm following Beer’s law. The UV–vis spectrum of the [Fe^III^(TCPP)] in MeOH showed a distinct absorption band at 414 nm (Figure 2b), which did not have an influence on the absorption band of gentian violet at 578 nm.

### 2.4. Degradation of Gentian Violet

The stock solution of gentian violet with a concentration of 10 mM and [Fe^III^(TCPP)] with a concentration of 0.315 mM in MeOH were prepared, which were kept for further use. It should be noted that the stock solution of PhIO (10 mM) was reconfigured with each use.

#### 2.4.1. General Procedure for the Effect of PhIO Concentration on Degradation of Gentian Violet

In a typical experiment, 160 μL of the stock solution of [Fe^III^(TCPP)] and 50 μL of the stock solution of gentian violet were put in 10 mL of MeOH to give a final concentration of 0.005 mM [Fe^III^(TCPP)] and 0.05 mM gentian violet. Then, 1 equiv, 2 equiv, 3 equiv, 4 equiv and 5 equiv of PhIO (relative to gentian violet, same as below) were added into the solution, respectively. The reaction mixture was stirred in a water bath at 303 K for 2.5 h. After that, the concentration of gentian violet in the resulting solution was determined by an ultraviolet–visible (UV–vis) spectrophotometer. Control experiments using [Fe^III^(TCPP)] or PhIO alone in gentian violet solution were performed in parallel. Reactions were performed at least in triplicate, and the data reported represent the average of these reactions.

#### 2.4.2. General Procedure for the Effect of Reaction Time on Degradation of Gentian Violet

In a typical experiment, 240 μL of the stock solution of [Fe^III^(TCPP)] and 75 μL of the stock solution of gentian violet were put in 15 mL of MeOH, and then 1–5 equiv of PhIO were added into this solution, respectively. Then, the reaction mixture was stirred in a water bath at 303 K. A total of 2 mL of each solution was taken at 0.5 h, 1 h, 1.5 h, 2 h and 2.5 h, respectively, to measure the UV–vis spectra. Reactions were performed at least in triplicate, and the data reported represent the average of these reactions.

## 3. Results and Discussion

**Figure 3:**
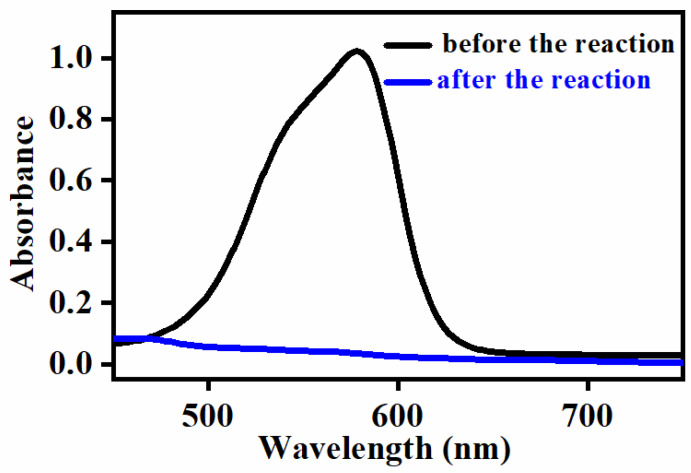
UV–vis spectral change in gentian violet before degradation (black line) and after degradation (blue line). Conditions: gentian violet 0.05 mM, [Fe^III^(TCPP)] 0.005 mM, PhIO 5 equiv, solvent MeOH, 303 K and reaction time 2.5 h.

The UV–vis spectra of the gentian violet and [Fe^III^(TCPP)] in MeOH exhibited a distinct absorption band at 578 nm and 414 nm, respectively, which indicated that their absorption peaks had no effect on each other. Thus, using UV–vis spectroscopy to monitor the concentration changes in the gentian violet was a suitable method. The degradation of the gentian violet was examined at fixed concentrations of catalyst [Fe^III^(TCPP)] and gentian violet dye in MeOH at 303 K using *m*-CPBA and PhIO as the oxidant, respectively, at preliminary experiments. No spectral change was observed when using 5 equiv of *m*-CPBA and reacting for 2.5 h. However, the color of the reaction solution changed from purple to colorless when using 5 equiv of PhIO and reacting for 2.5 h (Figure 3). Therefore, herein, PhIO and [Fe^III^(TCPP)] were employed as the oxidant and catalyst, respectively, for the degradation of the gentian violet.

**Figure 4:**
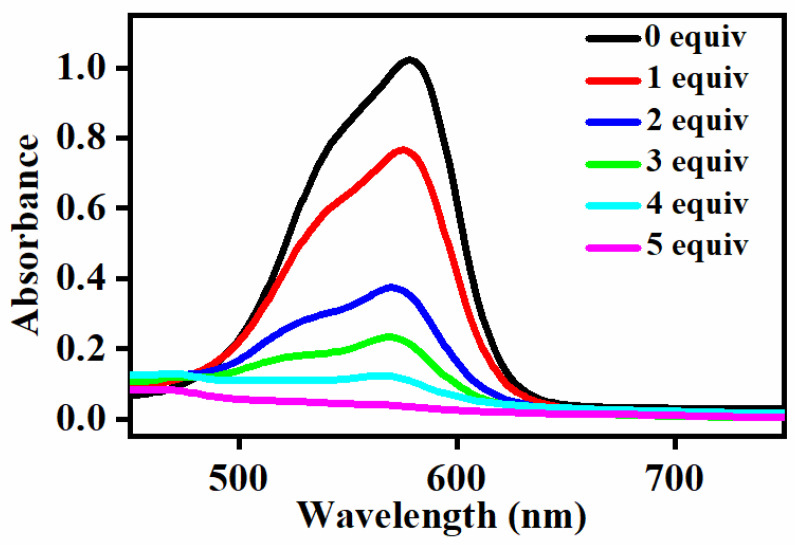
UV–vis spectral change observed in the degradation of gentian violet by various concentrations of PhIO. Conditions: gentian violet 0.05 mM; [Fe^III^(TCPP)] 0.005 mM; PhIO 0, 1, 2, 3, 4, 5 equiv; solvent MeOH; 303 K and reaction time 2.5 h.

The effect of PhIO concentration on the degradation of the gentian violet was first studied at fixed concentrations of [Fe^III^(TCPP)] and gentian violet in the [Fe^III^(TCPP)]/PhIO system. A typical set of PhIO concentration-dependent spectral absorptions recorded for the [Fe^III^(TCPP)]-catalyzed degradation of gentian violet in MeOH at 303 K is shown in Figure 4. The absorbance band at 578 nm, which was due to the gentian violet, decreased when increasing the concentration of PhIO, and it provided a 98% degradation efficiency when adding 5 equiv of PhIO (Table 1). The absorbance at 414 nm, which was due to the [Fe^III^(TCPP)], exhibited almost no change (Supplementary Materials Figure S1), which indicated that the catalyst [Fe^III^(TCPP)] was stable during the degradation of the gentian violet. No spectral changes were observed in the absence of [Fe^III^(TCPP)] or PhIO (Supplementary Materials, Figure S2). The spectral measurements confirmed that the presence of both [Fe^III^(TCPP)] and PhIO was essential for the degradation of the gentian violet and a high concentration of PhIO was beneficial in improving the degradation efficiency.

**Figure 5:**
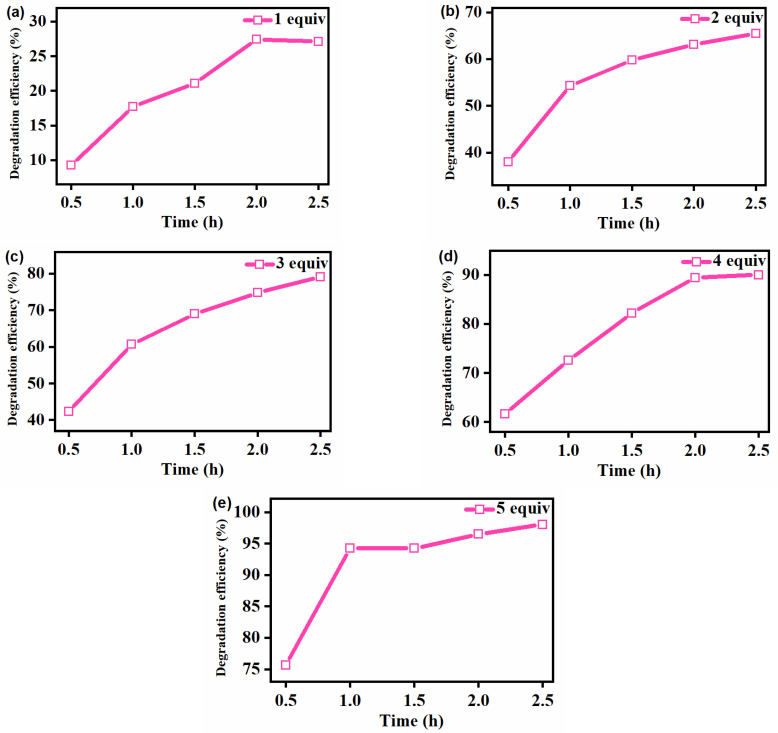
Effect of reaction time on the [Fe^III^(TCPP)]-catalyzed degradation of gentian violet by 1 equiv (**a**), 2 equiv (**b**), 3 equiv (**c**), 4 equiv (**d**) and 5 equiv (**e**) of PhIO. Conditions: gentian violet 0.05 mM; [Fe^III^(TCPP)] 0.005 mM; solvent MeOH and 303 K.

The effect of the reaction time on the degradation of the gentian violet was further examined at fixed concentrations of [Fe^III^(TCPP)] and gentian violet in the [Fe^III^(TCPP)]/PhIO system, and the results are presented in Figure 5, Table 2 and Figures S3–S6 in the Supplementary Materials. As shown in Figure 5 and Table 2, when using 1 equiv of PhIO, the degradation rate was the slowest and the degradation efficiency of gentian violet was the lowest compared with that of 2–5 equiv of PhIO. The degradation efficiency was basically unchanged after 2 h due to the insufficient amount of oxidant. When using 2 equiv and 3 equiv oxidants, although the corresponding degradation efficiency of gentian violet increased, the degradation rates were slow, and the degradation efficiency was only 65% and 79% after reacting for 2.5 h, respectively. When 4 equiv oxidant was used, with the increase in the reaction time, the degradation efficiency of gentian violet increased to 90%, but it remained almost unchanged after 2 h. When using 5 equiv oxidant, the degradation efficiency of gentian violet reached 75% within 0.5 h and 94% within 1 h, showing a remarkable degradation of gentian violet. The degradation products of the gentian violet produced by the [Fe^III^(TCPP)]/PhIO system after color removal were detected by GC-MS (Supplementary Materials, Figure S7). However, only iodobenzene (PhI) with a molecular weight of 204 was shown in the GC-MS results, which came from the reduction of PhIO. No product of the gentian violet was observed, which may have been due to the formation of high-boiling-point products, which could not be detected by GC-MS.

**Figure 6:**
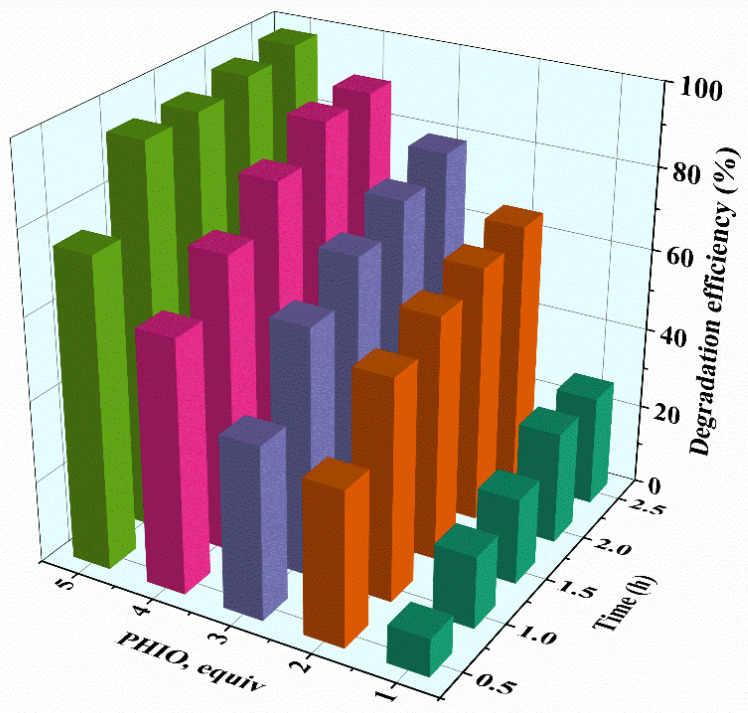
Degradation efficiency for the [Fe^III^(TCPP)]-catalyzed degradation of gentian violet by various concentrations of PhIO under various reaction times. Conditions: gentian violet 0.05 mM; [Fe^III^(TCPP)] 0.005 mM; solvent MeOH and 303 K.

The above experimental results are summarized in Figure 6, which indicated that with the increase in PhIO concentration and reaction time, the degradation efficiency of gentian violet increased, and it was up to more than 90% within 1 h when using 5 equiv of PhIO, showing a high catalytic degradation efficiency of the [Fe^III^(TCPP)]/PhIO system. Generally, the iron(IV)-oxo porphyrin π-cation radical species [Fe^IV^(O)(porph^+•^)] was proposed as the active species, which plays a major role in the catalytic oxidation process of iron porphyrin. Kong et al. proposed that an iron(IV)-oxo porphyrin π-cation radical, generated from the heterolytic cleavage of the O-O bond in H_2_O_2_ catalyzed by hemin, may play a major role in dye degradation [62]. Meyer et al. investigated the oxidative degradation of azo dyes by [Fe^III^(tmpyp)] and *m*-CPBA in aqueous solution at room temperature and suggested that [Fe^IV^(O)(tmpyp^+•^)] was capable of oxidatively breaking down the naphthalene core of azo dyes [52]. Thus, based on the previously suggested mechanisms [52,62] and the data obtained from the various experiments described above in the [Fe^III^(TCPP)]/PhIO system, it was assumed that the reaction between [Fe^III^(TCPP)] and PhIO generated a transient intermediate [Fe^IV^(O)(TCPP^+•^)], which rapidly interacted with gentian violet to make the gentian violet degrade and regenerate the catalyst [Fe^III^(TCPP)].

## 4. Conclusions

Various methods have been developed and widely applied for the degradation of gentian violet. However, these traditional methods have certain limitations. The traditional physical method is inefficient for eliminating dyes, especially from dilute solutions. The photo-oxidation method is relatively expensive and not appropriate for the treatment of large flows. Microbial treatment is considered to be an economical and feasible method without secondary pollution, yet biodegradation has the disadvantages of being unstable and time-consuming. Metalloporphyrins, as a representative model of cytochrome P450, have unparalleled catalytic specificity and efficiency, and they can be used as a catalyst to overcome the disadvantages of the above methods used to degrade dyes. In this work, a ferric porphyrin complex, [Fe^III^(TCPP)], as a cytochrome P450 model, was investigated for its potential to degrade dyes, where [Fe^III^(TCPP)] in combination with an oxidant, PhIO, was used for the degradation of the dye gentian violet. UV–vis absorption spectroscopy was used to monitor the concentration changes in the gentian violet via its UV–vis spectral changes. The [Fe^III^(TCPP)]/PhIO system provided a high catalytic degradation efficiency, where the degradation efficiency reached more than 90% in 1 h when using 5 equiv of PhIO at 303 K. Thus, as a cytochrome P450 model, iron porphyrins can play a great role in the degradation of dyes, which provides an efficient approach for the treatment of dye-containing wastewater.

## Figures and Tables

**Table 1:** [Fe^III^(TCPP)]-catalyzed degradation^1^ of gentian violet by PhIO.

Catalyst	PhIO (equiv)	Degradation Efficiency (%)
[Fe^III^(TCPP)]	-	0
-	5	0
[Fe^III^(TCPP)]	1	27
[Fe^III^(TCPP)]	2	65
[Fe^III^(TCPP)]	3	79
[Fe^III^(TCPP)]	4	90
[Fe^III^(TCPP)]	5	98

^1^ Conditions: gentian violet 0.05 mM; [Fe^III^(TCPP)] 0.005 mM; PhIO 0, 1, 2, 3, 4, 5 equiv; solvent MeOH; 303 K and reaction time 2.5 h.

**Table 2:** [Fe^III^(TCPP)]-catalyzed degradation ^1^ of gentian violet by various concentrations of PhIO under various reaction times.

Time (h)	Degradation Efficiency (%)				
	PhIO 1 equiv	PhIO 2 equiv	PhIO 3 equiv	PhIO 4 equiv	PhIO 5 equiv
0.5	9	38	42	61	75
1.0	18	54	60	72	94
1.5	21	60	69	82	94
2.0	27	63	74	89	96
2.5	27	65	79	90	98

^1^ Conditions: gentian violet 0.05 mM; [Fe^III^(TCPP)] 0.005 mM; PhIO; solvent MeOH and 303 K.

## Data Availability

Not applicable.

## Supplementary Materials

The following supporting information can be downloaded at: https://www.mdpi.com/article/10.3390/molecules27227948/s1, Figure S1: UV-visible absorption spectra of [Fe^III^(TCPP)] (blue line), the mixture of gentian violet and [Fe^III^(TCPP)] (red line), and the solution after the degradation of crystal violet by the [Fe^III^(TCPP)]/PhIO system (pink line); Figure S2: UV-visible absorption spectra of gentian violet (0.05 mM, blue line); the mixture of gentian violet (0.05 mM) and [Fe*III*(TCPP)] (red line); the solution of gentian violet (0.05 mM) and PhIO (5 equiv) after stirring 2.5 h (pink line); and the solution of gentian violet (0.05 mM) and PhIO (5 equiv) in the presence of [Fe^III^(TCPP)] after stirring 2.5 h (cyan line); Figure S3: UV−vis spectral change observed in the degradation of gentian violet by various concentrations of PhIO. Conditions: gentian violet 0.05 mM; [Fe^III^(TCPP)] 0.005 mM; PhIO 0, 1, 2, 3, 4, 5 equiv; solvent MeOH; 303 K; reaction time 0.5 h; Figure S4: UV−vis spectral change observed in the degradation of gentian violet by various concentrations of PhIO. Conditions: gentian violet 0.05 mM; [Fe^III^(TCPP)] 0.005 mM; PhIO 0, 1, 2, 3, 4, 5 equiv; solvent MeOH; 303 K; reaction time 1 h; Figure S5: UV−vis spectral change observed in the degradation of gentian violet by various concentrations of PhIO. Conditions: gentian violet 0.05 mM; [Fe^III^(TCPP)] 0.005 mM; PhIO 0, 1, 2, 3, 4, 5 equiv; solvent MeOH; 303 K; reaction time 1.5 h; Figure S6: UV−vis spectral change observed in the degradation of gentian violet by various concentrations of PhIO. Conditions: gentian violet 0.05 mM; [Fe^III^(TCPP)] 0.005 mM; PhIO 0, 1, 2, 3, 4, 5 equiv; solvent MeOH; 303 K; reaction time 2 h; Figure S7: GC-MS analysis of the degradation products of crystal violet degraded by the [Fe^III^(TCPP)]/PhIO system after color removal. (a) Gas chromatogram; (b) mass spectra for the peak with RT value of 5.028 min.

## References

[1] Reddy P.A.K., Reddy P.V.L., Kwon E., Kim K., Akter T., Kalagara S. (2016). Recent advances in photocatalytic treatment of pollutants in aqueous media. Environ. Int..

[2] Zhang X., Sun R., Sun S., Ren F., Chen X., Wu L., Xing R. (2019). Metal-free organic optoelectronic molecule as a highly efficient photocatalyst for the degradation of organic pollutants. ACS Omega.

[3] Dapaah M.F., Niu Q., Yu Y., You T., Liu B., Cheng L. (2022). Efficient persistent organic pollutant removal in water using MIL-metal-organic framework driven Fenton-like reactions: A critical review. Chem. Eng. J..

[4] Varjani S., Rakholiya P., Yong Ng H., You S., Teixeira J.A. (2020). Microbial degradation of dyes: An overview. Bioresour. Technol..

[5] Azmi W., Sani R.K., Banerjee U.C. (1998). Biodegradation of triphenylmethane dyes. Enzym. Microb. Technol..

[6] Parshetti G., Parshetti S., Telke A., Kalyani D., Doong R., Govindwar S.-P. (2011). Biodegradation of Crystal Violet by Agrobacterium radiobacter. J. Environ. Sci..

[7] Alharbi O.M.L., Basheer A.A., Khattab R.A., Ali I. (2018). Health and environmental effects of persistent organic pollutants. J. Mol. Liq..

[8] Ember E., Rothbart S., Puchta R., van Eldik R. (2009). Metal ion-catalyzed oxidative degradation of Orange II by H_2_O_2_. High catalytic activity of simple manganese salts. New J. Chem..

[9] Shanker U., Rani M., Jassal V. (2017). Degradation of hazardous organic dyes in water by nanomaterials. Environ. Chem. Lett..

[10] Rothbart S., Ember E., van Eldik R. (2010). Comparative study of the catalytic activity of [Mn^II^(bpy)_2_Cl_2_] and [Mn_2_^III/IV^(μ-O)_2_(bpy)_4_] (ClO_4_)_3_ in the H_2_O_2_ induced oxidation of organic dyes in carbonate buffered aqueous solution. Dalton Trans..

[11] Nidheesh P.V., Zhou M., Oturan M.A. (2018). An overview on the removal of synthetic dyes from water by electrochemical advanced oxidation processes. Chemosphere.

[12] Fu H.-R., Zhao Y., Xie T., Han M.-L., Ma L.-F., Zang S.-Q. (2018). Stable dye-encapsulated indium–organic framework as dual-emitting sensor for the detection of Hg^2+^/Cr_2_O_7_^2−^ and a wide range of nitro-compounds. J. Mater. Chem. C.

[13] Fu H.-R., Yan L.-B., Wu N.-T., Ma L.-F., Zang S.-Q. (2018). Dual-emission MOF⊃dye sensor for ratiometric fluorescence recognition of RDX and detection of a broad class of nitro-compounds. J. Mater. Chem. A.

[14] Qin J.-H., Qin W.-J., Xiao Z., Yang J.-K., Wang H.-R., Yang X.-G., Li D.-S., Ma L.-F. (2021). Efficient Energy-Transfer-Induced High Photoelectric Conversion in a Dye-Encapsulated Ionic Pyrene-Based Metal–Organic Framework. Inorg. Chem..

[15] Yang X.-G., Qin J.-H., Huang Y.-D., Zhai Z.-M., Ma L.-F., Yan D. (2020). Highly enhanced UV-vis-NIR light harvesting and photoelectric conversion of a pyrene MOF by encapsulation of the D–p–A cyanine dye. J. Mater. Chem. C.

[16] Wang Y., Wang S., Li X., Bai P., Yan W., Yu J. (2020). Layered Inorganic Cationic Frame-works beyond Layered Double Hydroxides (LDHs): Structures and Applications. Eur. J. Inorg. Chem..

[17] Nguyen T.L., Dinh Quoc V., Nguyen T.L., Le T.T.T., Dinh T.K., Nguyen V.T., Nguyen P.H. (2021). Visible-light-driven SO_4_^2-^/TiO_2_ photocatalyst synthesized from Binh Dinh (Vietnam) ilmenite ore for Rhodamine B degradation. J. Nanomater..

[18] Kumar A.P., Bilehal D., Tadesse A., Kumarc D. (2021). Photocatalytic degradation of organic dyes: Pd-γ-Al_2_O_3_ and PdO-γ-Al_2_O_3_ as potential photocatalysts. RSC Adv..

[19] Hariri R., Dehghanpour S., Sohrabi S. (2020). Facile ultrasonic synthesis of zirconium based porphyrinic MOFs for enhanced adsorption performance towards anionic and mixed dye solutions. J. Inorg. Organomet. Polym. Mater..

[20] Ahmed M.B., Zhou J.L., Ngo H.H., Guo W., Thomaidis N.S., Xu J. (2017). Progress in the biological and chemical treatment technologies for emerging contaminant removal from wastewater: A critical review. J. Hazard. Mater..

[21] Anis S.F., Lalia B.S., Lesimple A., Hashaikeh R., Hilal N. (2022). Electrically conductive membranes for contemporaneous dye rejection and degradation. Chem. Eng. J..

[22] Yin H., Zhao J., Li Y.Q., Huang L.L., Zhang H., Chen L.H. (2020). A novel Pd decorated Polydopamine-SiO_2_/PVA electrospun nanofiber membrane for highly efficient degradation of organic dyes and removal of organic chemicals and oils. J. Clean. Prod..

[23] Lopes L.S., Vieira N., da Luz J.M.R., Marliane de Cássia S.S., Cardoso W.S., Kasuya M.C.M. (2020). Production of fungal enzymes in macaúba coconut and enzymatic degradation of textile dye. Biocatal. Agric. Biotechnol..

[24] Li H.H., Wang Y.T., Wang Y., Wang H.X., Sun K.K., Lu Z.M. (2019). Bacterial degradation of anthraquinone dyes. J. Zhejiang Univ. Sci. B.

[25] Lan J., Chen R., Duo F., Hu M., Lu X. (2022). Visible-Light Photocatalytic Reduction of Aryl Halides as a Source of Aryl Radicals. Molecules.

[26] Li T.-T., Dang L.-L., Zhao C.-C., Lv Z.-Y., Yang X.-G., Zhao Y., Zhang S.-H. (2021). A self-sensitized Co(II)-MOF for efficient visible-light-driven hydrogen evolution without additional cocatalysts. J. Solid State Chem..

[27] Dang L.-L., Zhang T.-T., Li T.-T., Chen T., Zhao Y., Zhao C.-C., Ma L.-F. (2022). Stable Zinc-Based Metal-Organic Framework Photocatalyst for Effective Visible-Light-Driven Hydrogen Production. Molecules.

[28] Zhao Y., Wang L., Fan N.-N., Han M.-L., Yang G.-P., Ma L.-F. (2018). Porous Zn(II)-Based Metal-Organic Frameworks Decorated With Carboxylate Groups Exhibiting High Gas Adsorption and Separation of Organic Dyes. Cryst. Growth Des..

[29] Dang L.-L., Zong D.-X., Lu X., Zhang T.-T., Chen T., Sun J.-L., Zhao J.-Z., Liu M.-Y., Liu S.-R. (2022). The Selective CO_2_ Adsorption and Photothermal Conversion Study of an Azo-Based Cobalt-MOF Material. Molecules.

[30] Wu C.-D., Chen K. (2019). Designed fabrication of biomimetic metal–organic frameworks for catalytic applications. Coord. Chem. Rev..

[31] Lu X., Wang S., Qin J.-H. (2022). Isolating Fe-O_2_ intermediates in dioxygen activation by iron porphyrin complexes. Molecules.

[32] Zaragoza J.P.T., Goldberg D.P. (2018). Dioxygen binding and activation mediated by transition metal porphyrinoid complexes. Dioxygen-Dependent Heme Enzymes.

[33] Shah N.S., He X., Khan J.A., Khan H.M., Boccelli D.L., Dionysiou D.D. (2015). Comparative studies of various iron-mediated oxidative systems for the photochemical degradation of endosulfan in aqueous solution. J. Photochem. Photobiol. A.

[34] Hodges G.R., Smith J.R.L., Oakes J. (1998). Mechamism of oxidation of azo dyes by a sterically hindered anionic oxoiron(IV) porphyrin in aqueous solution. J. Chem. Soc. Perkin Trans..

[35] Zucca P., Rescigno A., Pintus M., Rinaldi A.C., Sanjust E. (2012). Degradation of textile dyes using immobilized lignin peroxidase-like metalloporphines under mild experimental conditions. Chem. Cent. J..

[36] Hodges G.R., Smith J.R.L., Oakes J. (1999). The oxidation of azo dyes by peroxy acids and tert-butyl hydroperoxide in aqueous solution catalysed by iron(III) 5,10,15,20-tetra (2,6-dichloro-3-sulfonatophenyl) porphyrin: Product studies and mechanism. J. Chem. Soc. Perkin Trans..

[37] Emmert F.L., Thomas J., Hon B., Gengenbach A.J. (2008). Metalloporphyrin catalyzed oxidation of methyl yellow and related azo compounds. Inorg. Chim. Acta.

[38] Barros V.P., Assis M.D. (2013). Iron porphyrins as biomimetical models for disperse azo dye oxidation. J. Braz. Chem. Soc..

[39] Saha T.K., Frauendorf H., John M., Dechert S., Meyer F. (2013). Efficient oxidative degradation of azo dyes by a water-soluble manganese porphyrin catalyst. ChemCatChem.

[40] Tokuda J., Ohura R., Iwasaki T., Takeuchi Y., Kashiwada A., Nango M. (1999). Decoloration of azo dyes by hydrogen peroxide catalyzed by water-soluble manganese porphyrins. Text. Res. J..

[41] Nango M., Iwasaki T., Takeuchi Y., Kurono Y., Tokuda J., Oura R. (1998). Peroxide decoloration of azo dyes catalyzed by polyethylene glycol-linked manganese halogenated porphyrins. Langmuir.

[42] Nakamura J., Oura R., Nango M. (2008). Peroxide decoloration of azo dye catalyzed by manganese porphyrin derivatives in non-aqueous solvent. Text. Res. J..

[43] Groves J.T., Stern M.K. (1987). Olefin epoxidation by manganese (IV) porphyrins: Evidence for two reaction pathways. J. Am. Chem. Soc..

[44] Umile T.P., Groves J.T. (2011). Catalytic generation of chlorine dioxide from chlorite using a water-soluble manganese porphyrin. Angew. Chem. Int. Ed..

[45] Nam W., Kim I., Lim M.H., Choi H.J., Lee J.S., Jang H.G. (2002). Isolation of an oxomanganese(V) porphyrin intermediate in the reaction of a manganese (III) porphyrin complex and H_2_O_2_ in aqueous solution. Chem.-Eur. J..

[46] Umile T.P., Wang D., Groves J.T. (2011). Dissection of the mechanism of manganese porphyrin-catalyzed chlorine dioxide generation. Inorg. Chem..

[47] Hicks S.D., Petersen J.L., Bougher C.J., Abu-Omar M.M. (2011). Chlorite dismutation to chlorine dioxide catalyzed by a water-soluble manganese porphyrin. Angew. Chem. Int. Ed..

[48] Arunkumar C., Lee Y.-M., Lee J.Y., Fukuzumi S., Nam W. (2009). Hydrogen-atom abstraction reactions by Manganese (V)-and Manganese (IV)-oxo porphyrin complexes in aqueous solution. Chem.-Eur. J..

[49] Groves J.T., Stern M.K. (1988). Synthesis, characterization, and reactivity of oxomanganese(IV) porphyrin complexes. J. Am. Chem. Soc..

[50] Rodgers K.R., Goff H.M. (1987). Detection of high-valent intermediates in the chlorine (I) oxidation of (porphinato) manganese(III) complexes. J. Am. Chem. Soc..

[51] Latifi R., Tahsini L., Karamzadeh B., Safari N., Nam W., De Visser S.P. (2011). Manganese substituted Compound I of cytochrome P450 biomimetics: A comparative reactivity study of Mn^V^-oxo versus Mn^IV^-oxo species. Arch. Biochem. Biophys..

[52] Saha T.K., Frauendorf H., Meyer F. (2021). Oxidative degradation of azo dyes in aqueous solution by water-soluble Iron porphyrin catalyst. Eur. J. Inorg. Chem..

[53] Ling B., Wang Y.G., Mi R., Wang D., Chen H.Q., Li X.H., Zhang Y., Wang L. (2022). Multimodal imaging and synergetic chemodynamic/photodynamic therapy achieved using an NaGdF_4_, Yb, Er@NaGdF_4_, Yb, Tm@NaYF_4_@Fe-MOFs nanocomposite. Chem. Asian J..

[54] Wu C.Y., Wu X.C., Hou F., Wu L., Liu G.X. (2022). An ultrasensitive electrochemical aptasensor based on Pd@PCN-222 as a signal probe coupled with exonuclease III-assisted cycling amplification for the detection of ochratoxin A. Food Control.

[55] Mishra J., Pattanayak D.S., Das A.A., Mishra D.K., Rath D., Sahoo N.K. (2019). Enhanced photocatalytic degradation of cyanide employing Fe-porphyrin sensitizer with hydroxyapatite palladium doped TiO_2_ nanocomposite system. J. Mol. Liq..

[56] Zhang K., Goswami S., Noh H., Lu Z.Y., Sheridan T., Duan J.X., Dong W., Hupp J.T. (2022). An iron-porphyrin grafted metal-organic framework as a heterogeneous catalyst for the photochemical reduction of CO_2_. Photochem. Photobiol..

[57] Cong M.Y., Chen X.Y., Xia K., Ding X., Zhang L.L., Jin Y., Gao Y., Zhang L.X. (2021). Selective nitrogen reduction to ammonia on iron porphyrin-based single-site metal-organic frameworks. J. Mater..

[58] Dang L.-L., Li T.-T., Zhang T.-T., Zhao Y., Chen T., Gao X., Ma L.-F., Jin G.-X. (2022). Highly selective synthesis and near-infrared photothermal conversion of metalla-Borromean ring and [2]catenane assemblies. Chem. Sci..

[59] Zhu P., Xu Z., Cai L., Chen J. (2021). Porphyrin iron-grafted mesoporous silica composites for drug delivery, dye degradation and colorimetric detection of hydrogen peroxide. Nanoscale Res. Lett..

[60] Dauban P., Sanière L., Tarrade A., Dodd R.H. (2001). Copper-catalyzed nitrogen transfer mediated by iodosylbenzene PhI=O. J. Am. Chem. Soc..

[61] Lucas H.J., Kennedy E.R. (1955). Organic Syntheses Collective.

[62] Yan M., Xie H., Zhang Q., Qu H., Shen J., Kong J. (2016). Hemin Based Biomimetic Oxidative Degradation of Acid Orange 7. J. Mater. Sci. Chem. Eng..

