# Supplementary material for: Iron Porphyrin as a Cytochrome P450 Model for the Degradation of Dye
Source: Molecules. 2022 Nov 17;27(22):7948. doi: 10.3390/molecules27227948 (PMC9696844; doi:10.3390/molecules27227948)
Supplement: Supplementary file 1 [file molecules-27-07948-s001.zip › molecules-1989253-supplementary.pdf]

Supplementary Materials

Iron Porphyrin as a Cytochrome P450 Model for the Degradation of Dye

Dan-Dan Ren [1,2,†], Xiaoyan Lu [2,†,*], Li-Ping Zhou [2], Huanghongjun Tian [2], Shuang Wang [2], Lu-Fang Ma [1,2,*], Dong-Sheng Li [1]

[1] College of Materials and Chemical Engineering, China Three Gorges University, Yichang, 443002, China

[2] Henan Key Laboratory of Function-Oriented Porous Materials, College of Chemistry and Chemical Engineering, Luoyang Normal University, Luoyang, 471934, China

* Correspondence: zklxiaoyan@163.com (X.L.); mazhuxp@126.com (L.-F.M.)

† These authors contributed equally to this work.

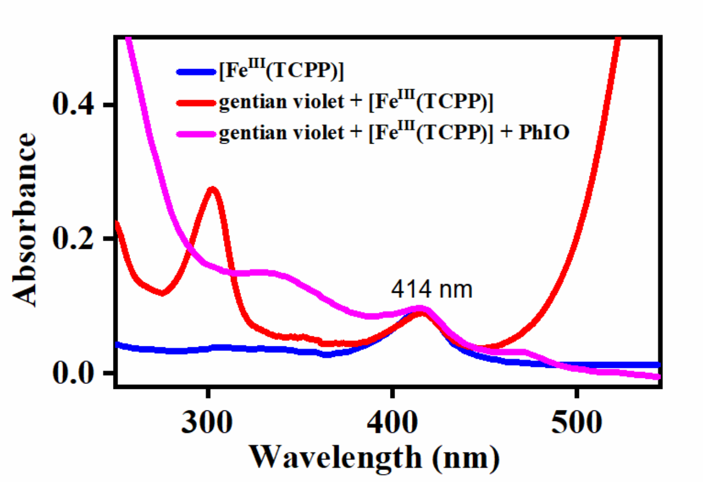

Figure S1. UV-visible absorption spectra of [Fe^{III}(TCPP)] (blue line), the mixture of gentian violet and [Fe^{III}(TCPP)] (red line), and the solution after the degradation of crystal violet by the [Fe^{III}(TCPP)]/PhIO system (pink line).

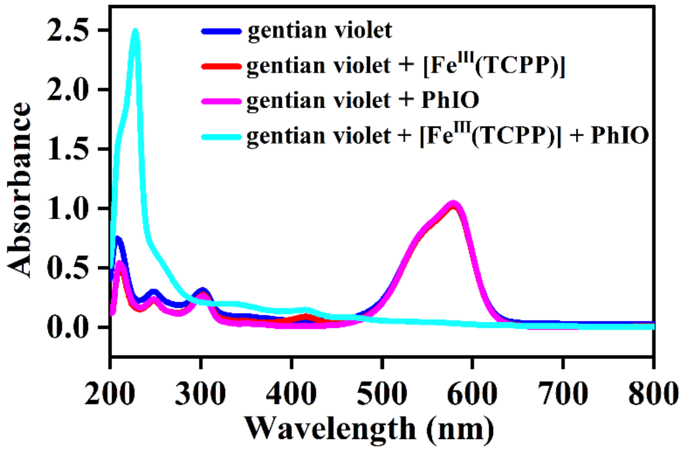

Figure S2. UV-visible absorption spectra of gentian violet (0.05 mM, blue line); the mixture of gentian violet (0.05 mM) and [Fe^{III}(TCPP)] (red line); the solution of gentian violet (0.05 mM) and PhIO (5 equiv) after stirring 2.5 h (pink line); and the solution of gentian violet (0.05 mM) and PhIO (5 equiv) in the presence of [Fe^{III}(TCPP)] after stirring 2.5 h (cyan line).

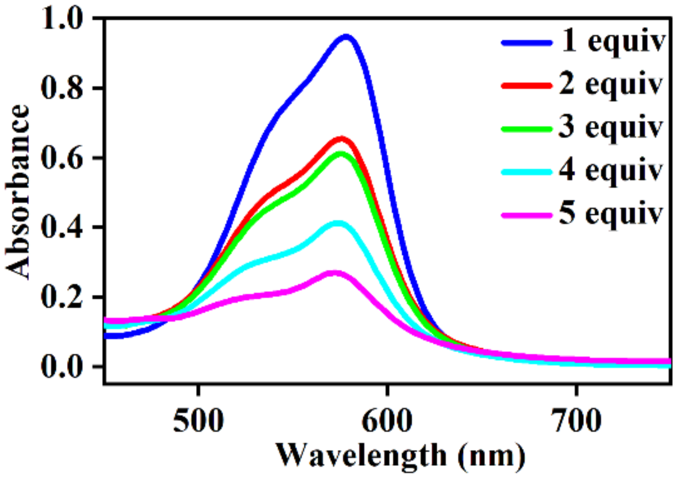

Figure S3. UV−vis spectral change observed in the degradation of gentian violet by various concentrations of PhIO. Conditions: gentian violet 0.05 mM; [Fe^{III}(TCPP)] 0.005 mM; PhIO 0, 1, 2, 3, 4, 5 equiv; solvent MeOH; 303 K; reaction time 0.5 h.

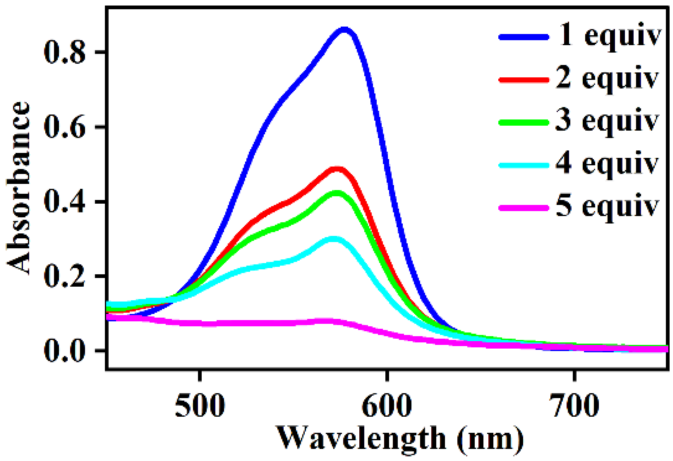

Figure S4. UV−vis spectral change observed in the degradation of gentian violet by various concentrations of PhIO. Conditions: gentian violet 0.05 mM; [Fe^{III}(TCPP)] 0.005 mM; PhIO 0, 1, 2, 3, 4, 5 equiv; solvent MeOH; 303 K; reaction time 1 h.

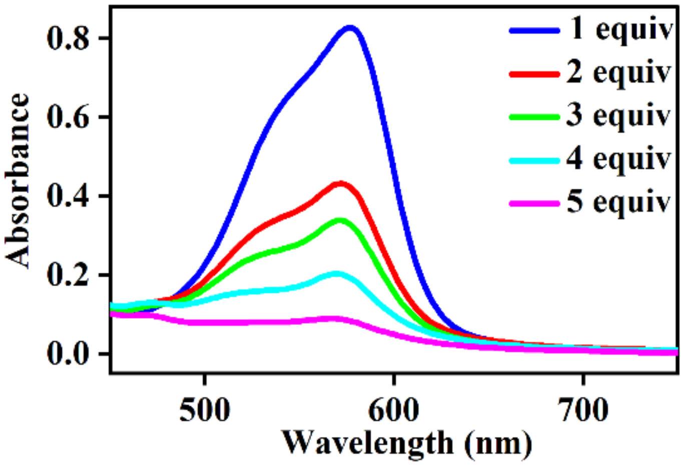

Figure S5. UV−vis spectral change observed in the degradation of gentian violet by various concentrations of PhIO. Conditions: gentian violet 0.05 mM; [Fe^{III}(TCPP)] 0.005 mM; PhIO 0, 1, 2, 3, 4, 5 equiv; solvent MeOH; 303 K; reaction time 1.5 h.

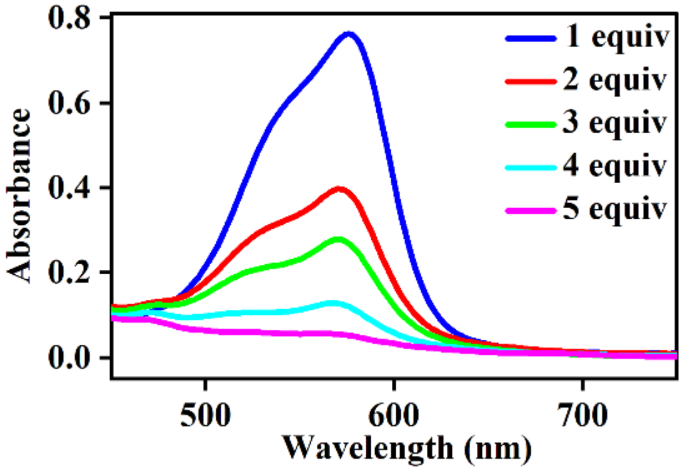

Figure S6. UV−vis spectral change observed in the degradation of gentian violet by various concentrations of PhIO. Conditions: gentian violet 0.05 mM; [Fe^{III}(TCPP)] 0.005 mM; PhIO 0, 1, 2, 3, 4, 5 equiv; solvent MeOH; 303 K; reaction time 2 h.

(a)

(b)

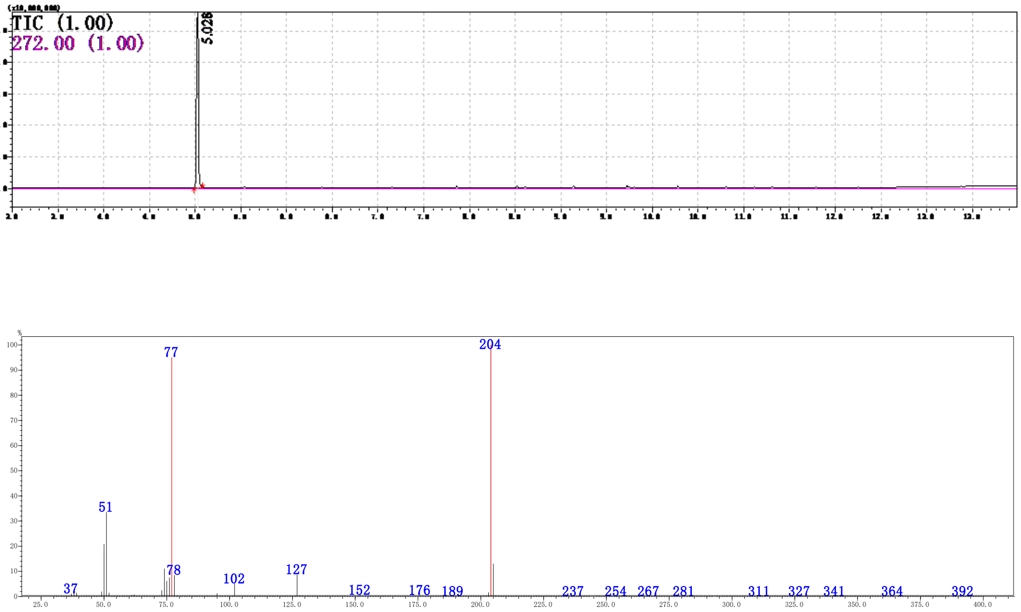

Figure S7. GC-MS analysis of the degradation products of crystal violet degraded by the [Fe^{III}(TCPP)]/PhIO system after color removal. (a) Gas chromatogram; (b) mass spectra for the peak with R_T value of 5.028 min.
